# *TP53* Mutation-Mediated Immune Evasion in Cancer: Mechanisms and Therapeutic Implications

**DOI:** 10.3390/cancers16173069

**Published:** 2024-09-03

**Authors:** Chuqi Wang, Jordan Yong Ming Tan, Nishtha Chitkara, Shruti Bhatt

**Affiliations:** 1 Department of Pharmacy & Pharmaceutical Sciences, National University of Singapore, Singapore 117559, Singapore; chuqiwang97@u.nus.edu (C.W.); jordantanym@u.nus.edu (J.Y.M.T.); 2 Duke-NUS Medical School, Singapore 169857, Singapore; e1404424@u.nus.edu

**Keywords:** p53, p53 mutation, immune evasion, mouse double minute 2, immunotherapy

## Abstract

**Simple Summary:**

p53 mutations are prevalent across a variety of human cancers and are regarded as a major obstacle in cancer therapy. These mutations can confer resistance to apoptosis and cell cycle arrest, contributing to multidrug resistance in tumors. Recent studies have uncovered important immunomodulatory functions of p53, but these functions are still underappreciated compared to its other well-known roles. This review aims to summarize the latest literature on immune evasion in p53-mutant tumors and explore the potential of targeting p53 to enhance anti-tumor immunity.

**Abstract:**

Mutation in p53 is the most frequent event in cancer development and a leading cause of cancer therapy resistance due to evasion of the apoptosis cascade. Beyond chemotherapies and radiation therapies, growing evidence indicates that p53-mutant tumors are resistant to a broad range of immune-based therapies, such as immune checkpoint inhibitors, chimeric antigen receptor (CAR) T, and hematopoietic stem cell transplantation (HSCT). This highlights the role of p53 mutations in driving immune evasion of tumor cells. In this review, we first summarize recent studies revealing mechanisms by which p53-mutant tumors evade immune surveillance from T cells, natural killer (NK) cells, and macrophages. We then review how these mutant tumor cells reshape the tumor microenvironment (TME), modulating bystander cells such as macrophages, neutrophils, and regulatory T (Treg) cells to foster immunosuppression. Additionally, we review clinical observations indicative of immune evasion associated with p53 loss or mutations. Finally, we discuss therapeutic strategies to enhance immune response in p53 wild-type (WT) or mutant tumors.

## 1. Introduction

**Figure 1:**
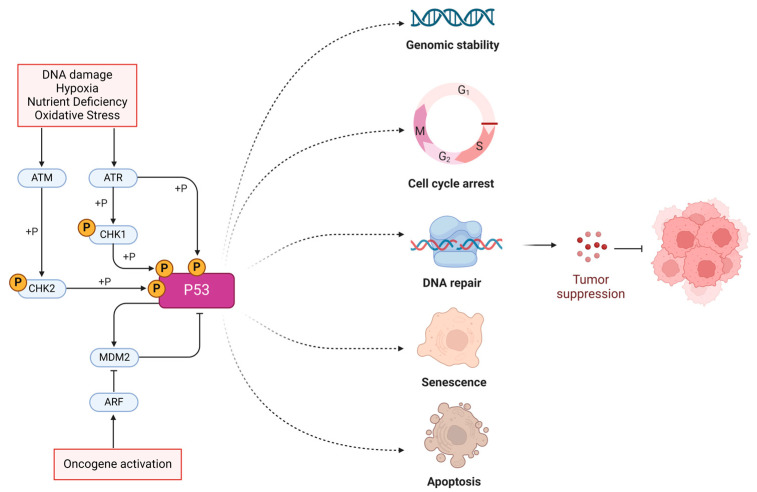
Molecular functions of p53. p53 is activated by cytotoxic stressors such as DNA damage, hypoxia, nutrient deficiency, oxidative stress, or oncogene activation via the inhibition of the MDM-2 pathway. p53 acts as a tumor suppressor, ensuring genomic stability within the cell by inducing cell cycle arrest, DNA repair mechanisms, senescence, or apoptosis, which consequently leads to effective tumor suppression.

*TP53* is a gene located on chromosome 17 that provides instructions for making the p53 protein. This protein plays a key role in regulating the cell cycle, maintaining genomic stability, and suppressing oncogenesis (Figure 1). The *TP53* gene is one of the most frequently mutated genes in human tumors. Estimates suggest that mutations in *TP53* are found in over 50% of all human tumors. The frequency of *TP53* mutations can also vary depending on the type of cancer, but its high mutation rate is a common characteristic across many cancer types, spanning both solid tumors and hematological malignancies [1,2,3]. The p53 protein functions as a “guardian of the genome” by monitoring DNA integrity, initiating cellular responses to DNA damage, and functioning as a tumor suppressor by triggering apoptosis in cells with damaged DNA or other abnormalities [4,5]. Upon activation by stress stimuli, the p53 protein is released from the MDM-2/4-p53 complex, accumulates in the cell, and regulates the transcription of over 500 genes directly and indirectly, thus affecting numerous cellular functions. Critically, p53 seizes cell cycle progression through the upregulation of p21 and induces apoptosis in irreparably damaged cells via the direct transcriptional activation of pro-apoptotic BH3-only proteins, such as PUMA and NOXA [6,7]. Therefore, a loss of function in p53 due to mutations leads to selective clonal advantage to bypass checkpoints that prevent uncontrolled proliferation of abnormal cells [8]. Certain p53 mutations predominantly manifest dominant-negative effects over its wild-type (WT) counterpart, suppressing any residual regulatory function of the latter [9]. Some mutations in *TP53* not only disrupt its normal functions but also endow the p53 protein with new, potentially harmful properties. These gain-of-function (GOF) mutations may account for 30% of all missense mutations of the *TP53* gene and can contribute to increased cancer progression, metastasis, and resistance to therapies [10,11].

Given p53’s critical role as a tumor suppressor, p53 alterations often correlate with poor clinical outcomes, frequently heralding a dismal prognosis [12]. These mutations lead to the expression of a structurally and conformationally abnormal p53 protein that is less effective at canonical tumor suppressive functions, coupled with the gain of new functions that further drive tumorigenesis. Specifically, it loses the ability to initiate apoptosis in cancer cells with mutated or damaged DNA, allowing these cells to survive and proliferate despite DNA damage induced by chemotherapeutic agents [13]. The loss of functional p53 also disrupts regulation of the cell cycle, particularly at the G1/S checkpoint, enabling cells with damaged DNA to proliferate. Furthermore, it can also bypass the induction of cellular senescence, a state of permanent cell cycle arrest, allowing cancer cells to evade the cytotoxic effects of chemotherapy. p53 also influences the tumor microenvironment, potentially creating a supportive microenvironment for tumor growth and resistance to chemotherapies [14]. By impairing these critical cellular processes, mutant p53 allows cancer cells to evade the cytotoxic effects of chemotherapy, thus accounting for poor clinical outcomes.

While p53 mutations have been extensively studied in the context of genomic instability and chemotherapy resistance, their role in modulating the immune response remains incompletely understood. Immunotherapy has revolutionized cancer treatment by leveraging the body’s immune system to target and eliminate tumor cells. However, p53-mutant cancers present unique challenges to this approach due to several factors contributing to immunotherapy resistance. Emerging evidence suggests that p53-mutant cancer cells exhibit unique immune evasion mechanisms that underscore the multifaceted role of p53 in modulating immune responses and shaping the TME [15,16,17]. Therefore, in this review, we delve into the complex interplay between p53 mutations in cancers and their consequent capacity for immune evasion. By shedding light on the influence of p53 mutations on the tumor microenvironment, we can better understand the emerging strategies designed to overcome the immune evasive capacity of p53-mutant cancers. We aim to summarize known evidence surrounding the clinical response of p53-mutant cancers to immunotherapy and elucidate the mechanistic underpinnings driving immune dysregulation in p53-mutant cancers. This exploration will provide valuable insights into the development of more effective cancer immunotherapies and potential avenues for improving clinical outcomes in patients with p53-mutant tumors.

## 2. *TP53* Gene Structure, Mutation Spectrum, and Etiology

The *TP53* gene is located on the short arm of chromosome 17 (17p13.1) and spans approximately 20 kilobases of DNA [18]. Structurally, *TP53* comprises 11 exons and 10 introns and encodes the p53 protein, which is a phosphoprotein consisting of 393 amino acids [19]. The exons are responsible for encoding the various functional domains of the p53 protein, including the transactivation domain, the DNA-binding domain, and the tetramerization domain. The sequence of exons in *TP53* includes several functional domains:

- Exon 1–2: Encodes the transactivation domain, which is responsible for activating the transcription of target genes.
- Exon 3–4: Contains sequences that contribute to the proline-rich region, which is important for the apoptotic function of p53.
- Exon 5–8: Encodes the DNA-binding domain, a crucial region that allows the p53 protein to bind to specific DNA sequences and regulate gene expression. This region is also the most frequently mutated in cancers.
- Exon 9: Encodes part of the oligomerization domain, which is essential for the tetramer formation of the p53 protein.
- Exon 10–11: Encodes the remaining part of the oligomerization domain and the C-terminal regulatory domain, which is involved in the regulation of p53’s activity.

Approximately 80% of *TP53* mutations are missense mutations that are primarily found in exons 5–8, which encode the DNA-binding domain. According to the Catalogue of Somatic Mutations in Cancer (COSMIC) database, the most frequently mutated sites across all tumor types include R175, G245, R248, R249, R273, and R282 [20]. In contrast, the introns play roles in gene regulation, mRNA stability, and alternative splicing, which can influence the protein’s functionality. Less commonly, *TP53* intronic polymorphisms were also discovered, which may regulate gene expression and affect susceptibility to cancer. For instance, earlier studies have indicated that a polymorphism in intron 6 is significantly linked to an increased risk of various cancers, such as gastrointestinal tumors, breast cancer, thyroid cancer, and ovarian cancer, and is associated with the notorious Li–Fraumeni syndrome. Common hotspot mutations in p53 and their respective phenotypes are summarized in Table 1.

The etiology of *TP53* gene mutations is influenced by a combination of genomic instability, environmental factors, and inherited genetic predispositions [31]. Genomic instability, often resulting from errors in DNA replication or repair, can lead to mutations in *TP53*. Environmental carcinogens, such as tobacco smoke, UV radiation, and certain chemicals, can further exacerbate this instability by directly causing DNA damage and introducing mutations into the *TP53* gene [32]. Germline mutations in *TP53*, as observed in disorders like the Li–Fraumeni syndrome, can predispose individuals to a higher risk of developing multiple cancers from birth [33]. Additionally, infections, like those caused by human papillomavirus (HPV), can interfere with p53 function and increase mutation rates [34]. Moreover, recent studies have revealed that p53 activity can be regulated at the post-translational level [35].

## 3. Mechanisms of Immune Evasion Caused by *TP53* Mutations

**Figure 2:**
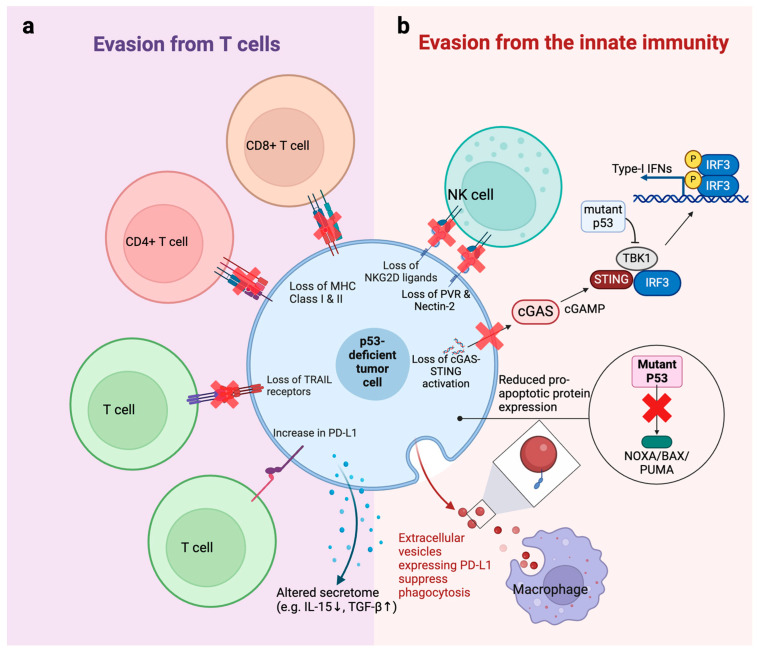
Mechanisms of immune evasion mediated by p53 mutation or deletion. (**a**) p53-deficient tumor cells employ multiple strategies to evade T cell-mediated killing, including downregulation of MHC Class I and II molecules, loss of TRAIL receptors, and upregulation of PD-L1 expression. (**b**) p53 mutations hinder the anti-tumor functions of the innate immunity. p53-mutant cells are evasive to NK cells by downregulation of NK-activating ligands (e.g., NKG2D ligands, PVR, and Nectin-2) and suppressing the cGAS-STING pathway. Additionally, p53 mutation leads to reduced expression of pro-apoptotic protein (NOXA/BAX/PUMA), which makes the tumor cells resistant to NK-mediated apoptosis. p53-mutant cells also release extracellular vesicles expressing PD-L1 to inhibit phagocytosis by macrophages, contributing to the immune evasion. Alterations in cancer cell secretome (e.g., reduced IL-15 and increased TGF-β) further suppress overall immune response.

Accumulating evidence from basic research suggests that p53-mutant tumors are more immune evasive compared to p53-WT tumors. In this section, we summarize the mechanisms of immune evasion from both the adaptive and innate immune systems driven by *TP53* mutations/deletion (Figure 2). The immunomodulatory effects of different p53 mutations are summarized in Table 2.

### 3.1. Evasion from T Cells

T cells are the most critical immune cells in surveilling and eliminating malignant transformed cells. In vivo CRISPR screening identified *TP53* as a top tumor suppressor gene in immunocompetent WT mice but not in SCID immunodeficient mice using the 4T1 breast tumor syngeneic model, suggesting that loss of *TP53* may contribute to evasion from the adaptive immune system [45]. Tumor cells present peptides derived from tumor-associated antigens or tumor-specific antigens on their surface in the context of major histocompatibility complex (MHC) class I molecules, which T cells recognize through their T cell receptors (TCRs). It has been shown that tumor cells could develop resistance to T cells by alterations in MHC molecules, making them less recognizable to cytotoxic T lymphocytes [46]. They can also upregulate immune checkpoints and soluble immunosuppressive molecules to contribute to evasion from T cells [47]. Evidence suggests that p53 mutations can cause T cell immune evasion by similar mechanisms (Figure 2a).

p53 can regulate immune-activating ligands on tumor cells. p53 has been shown to regulate MHC class I and II expression in various cancer types via diverse mechanisms. p53 can transcriptionally activate endoplasmic reticulum aminopeptidase 1 (ERAP1) expression [36]. ERAP1 is responsible for the final trimming of antigen precursors before loading into MHC class I proteins. Defects in ERAP1 result in changes in MHC class I expression and impaired T cell responses. Therefore, p53-null tumor cells have less MHC class I expression, highlighting the important role of p53 in the immune response [36]. The transporter associated with antigen processing (TAP) gene plays an essential role in MHC class I antigen presentation. Zhu et al. found that p53 induced surface MHC class 1 expression by upregulating TAP1 [48], which was also observed by another study [36]. Beyond MHC I, p53 was also shown to regulate MHC class II. Activating p53 by mouse double minute 2 (MDM2) inhibitors, a class of p53-activating agents, could upregulate MHC class II in p53-WT but not p53-knockdown (KD) acute myeloid leukemia (AML) cells, suggesting that the regulation of MHC II by MDM2 inhibitor is p53 dependent [49]. Apoptosis-inducing ligand receptor-1 and -2 (TRAIL-R1/2) plays a central role in the graft versus leukemia (GVL) effect during hematopoietic stem cell transplantation therapy [50]. p53 was reported to be a transcription factor for TRAIL-R1/2, which partially explains the immune evasion of p53-mutant AML cells [49].

In addition to immune-activating molecules, p53 can regulate immunosuppressive molecules in tumor cells, such as the immune checkpoint PD-L1 (CD274). Cortez et al. showed that WT p53 downregulated PD-L1 expression by increasing levels of miR-34 microRNA [37]. Another study demonstrated a similar regulatory mechanism via miR-320a along with miR-200a and miR-34a [51]. These findings suggest that p53 mutations could cause PD-L1 upregulation and, therefore, immune evasion.

Beyond the cell surface molecules mentioned above, *TP53* may regulate the secretome of tumor cells, which mediates immune activation or suppression. One study found that p53 induction by MDM2 inhibitor upregulated IL-15, which serves as an activator of anti-tumor CD8+ T cells and NK cells [52]. Another study showed that an AML cell line with *TP53* KD secreted more TGF-β to suppress T cell proliferation and cytotoxicity [53]. Whether other soluble factors are also regulated by p53 remains to be determined.

### 3.2. Evasion from the Innate Immune System

p53 mutation can drive immune evasion by disrupting immunosurveillance mechanisms within the innate immune system (Figure 2b). As an important member of the innate immune system, natural killer (NK) cells supplement the function of T cells by killing MHC-defective tumor cells [54]. Unlike T cells, NK cells do not require MHC. They recognize abnormal cells by a variety of activating and inhibitory receptors [55]. Studies showed that p53 is important in NK immune surveillance. p53 transcriptionally regulates ULBP1 and ULBP2 in tumor cells, which serve ligands for the NKG2D receptor on NK cells [42]. Inducing WT p53 but not mutant p53 in tumor cells increased recognition by NK cells characterized by higher IFN-γ expression and degranulation in NK cells, highlighting the importance of WT p53 in NK recognition. In addition to NKG2D ligands, ligands for DNAM1 are also modulated by p53 [56]. Veneziani et al. showed that p53 could activate the transcription of PVR and Nectin-2, which are ligands for DNAM1. Restoring p53 by MDM2 inhibitor Nutlin-3a increased PVR and Nectin-2 expression and sensitized neuroblastoma cells to NK adoptive immunotherapy [56]. On top of reduced NK recognition, tumor cells might acquire intrinsic resistance to NK cytotoxicity by reducing their sensitivity to apoptosis. A study showed that the mitochondrial apoptosis of tumor cells is vital for NK-mediated killing [57]. Mitochondrial apoptosis is regulated by a series of pro-apoptotic and anti-apoptotic proteins in the mitochondria. p53 is known to induce the expression of pro-apoptotic proteins Noxa [58], Bax [59], and Puma [6], thus making tumor cells more primed to mitochondrial apoptosis. Therefore, loss of p53 might also cause resistance to NK cells by reducing the sensitivity of cancer cells to apoptosis.

Macrophages serve as a part of the body’s first line of defense against pathogens but have a complex role in cancers. In antibody-based therapies, macrophages are extremely important anti-tumor effectors. They exert their anti-tumor effect via antibody-dependent cellular phagocytosis (ADCP) [60]. p53-null B cell malignancies were shown to have reduced phagocytosis by macrophages [38]. Mechanistically, p53-null cells produce more extracellular vesicles (EV) compared to p53-WT cells. These EV from p53-null cells carry higher PD-L1, which inhibits ADCP by macrophages. This study demonstrated that *TP53* mutations may cause resistance to ADCP and that PD-1/PD-L1 checkpoint inhibitors may be a good partner with therapeutic antibodies in treating p53-deficient B cell malignancies.

Additionally, the loss and mutation of p53 have also been reported to interfere with the function of the cGAS-STING cytosolic DNA sensing pathway. The cGAS-STING pathway is a critical mechanism of the innate immune system that detects cytosolic DNA, a signal often associated with viral infections or cellular damage [14,61]. Upon sensing DNA, the enzyme cGAS produces cyclic GMP-AMP (cGAMP), which activates the STING protein located in the endoplasmic reticulum. This activation triggers a signaling cascade involving TBK1 and IRF3, leading to the production of type I interferons and other cytokines that orchestrate the immune response and enhance the body’s defense against pathogens. Ghosh et al. has recently found that *TP53* promotes the degradation of cytosolic DNA exonuclease TREX1, causing cytoplasmic DNA accumulation and activation of the cGAS-STING pathway [39]. Consequently, the deletion of *TP53* results in a direct loss of cGAS-STING pathway activation, while mutant p53 proteins but not wild-type p53 have been found to bind to TANK-binding protein kinase 1 (TBK1) [44]. This abrogates the formation of a critical trimeric complex between TBK1, STING, and IRF3, which is necessary for the activation of the innate immune response via the cGAS-STING-TBK1-IRF3 pathway. As such, it is postulated that the loss or mutation of *TP53* results in escape from the innate immune system.

## 4. p53-Mutant Tumor Cells Create an Immunosuppressive TME to Promote Immune Evasion

**Figure 3:**
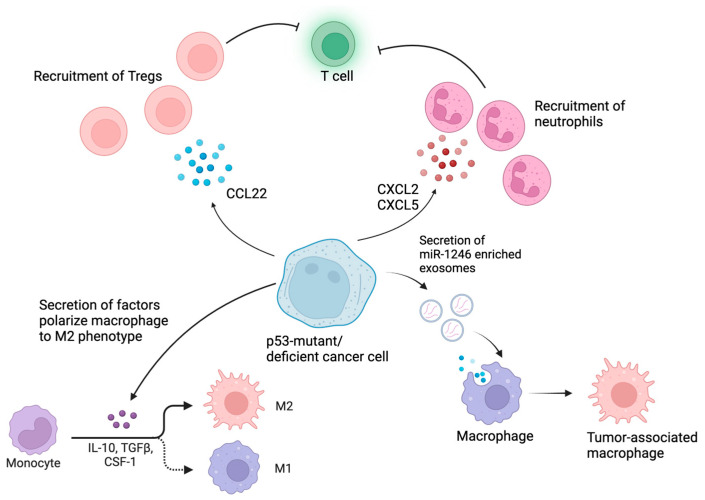
p53-mutant/deficient tumor cells reshape the TME to cause immunosuppression. p53-mutant tumor cells can release cytokines and exosomes to reprogram macrophages into immunosuppressive phenotypes. These mutant tumor cells can also recruit neutrophils and Tregs into the TME to suppress T cell functions.

The crosstalk between *TP53*-mutated cancer cells and the tumor microenvironment, particularly involving myeloid cells and Treg cells, plays a crucial role in tumor progression and immune evasion. Myeloid cells, including macrophages and neutrophils, exhibit phenotypic and functional alterations in the presence of *TP53*-mutated cancer cells [62]. These alterations often lead to an immunosuppressive microenvironment that facilitates tumor growth and metastasis (Figure 3).

### 4.1. p53 Mutations Reprogram Myeloid Cells in the TME into Immunosuppressive Phenotypes

Within the TME, macrophages can adopt different functional states, broadly categorized as M1-like (classically activated) or M2-like (alternatively activated). M2-like macrophages are often found to be correlated with immunosuppression and failure of immunotherapies in various types of cancer [63,64]. p53 mutations within cancer cells have been shown to skew the polarization of macrophages towards an M2-like phenotype [43,65]. This shift in polarization is often associated with an immunosuppressive microenvironment that promotes tumor growth, invasion, and metastasis. p53-mutated cancer cells may also secrete factors such as CSF-1 (colony-stimulating factor 1) [40], IL-10 (interleukin-10) [43], and TGF-β (transforming growth factor-beta) [53], which are known to polarize macrophages towards the M2 phenotype. Li et al. have also demonstrated that p53 suppresses M2 polarization through the inhibition of c-myc, a transcription factor that promotes M2 macrophage polarization [66]. Beyond disrupting the tumor-suppressive functions of WT p53, certain missense mutations confer GOF activities to mutant p53 (mutp53) proteins [11]. These GOF activities profoundly alter tumor cell behavior by influencing protein interactions and modulating transcriptional programs. As such, it has been shown that p53-mutant cancer cells can actively reprogram macrophages into a tumor-supportive and anti-inflammatory state. Specifically, colon cancer cells carrying GOF mutp53 selectively release exosomes enriched with miR-1246 [43]. Upon uptake by neighboring macrophages, these exosomes induce miR-1246-dependent reprogramming that favors cancer progression. The mutp53-reprogrammed tumor-associated macrophages (TAMs) exhibit enhanced immunosuppressive activity, characterized by increased levels of TGF-β. Another study showed that the GOF *TP53* R172H mutation drives an increase in CD11b+Ly6G+ neutrophils, accompanied by a reduction in CD3+ T cells, CD8+ T cells, and CD4+ T helper 1 cells [41].

### 4.2. p53-Mutant Cancers Recruit Tregs to Cause Immunosuppression

Tregs are a key component of the immunosuppressive network within the TME, helping cancer cells evade the immune system and promoting tumor progression [67]. They can be recruited to the tumor microenvironment by various chemokines, and these Tregs promote tumor growth via inhibition of anti-tumor cells such as cytotoxic CD8+ T cells. One study showed that p53 deficiency in prostate, ovarian, and pancreatic cancers increased the frequency of Tregs in the TME. This was demonstrated to be via the suppression of miR-34a, causing enhanced production of CCL22 and enhancing Treg recruitment to the TME [68]. When Tregs are recruited by CCL22, they have also been shown to be selectively activated in the lymphoid infiltrates surrounding breast tumors, which is associated with an adverse clinical outcome in primary breast cancer patients [69]. Blagih et al. found that p53-null tumor cells had an accumulation of Tregs, accompanied by a lower CD4+ T helper 1 (Th1) and CD8+ T cell response in mouse models [62]. Tregs from AML patients with *TP53* mutations have also been shown to exhibit distinct metabolic characteristics. These Tregs display an enrichment of gene sets linked to glycolysis, fatty acid metabolism, and oxidative phosphorylation relative to controls [70]. This indicates that p53-mutant cancers possibly influence Tregs within the tumor microenvironment to enhance their energy production using both glucose and fatty acids in the context of AML.

Overall, the crosstalk between p53-mutant cancer cells and the tumor microenvironment, particularly involving myeloid cells and Treg cells, creates a conducive immunosuppressive niche that promotes tumor growth, invasion, and resistance to immunotherapy. Understanding these interactions is essential for developing novel therapeutic strategies to target p53-mutant tumors and overcome immune evasion mechanisms.

## 5. Clinical Evidence of Immune Evasion in p53-Mutant Cancers

Mutations in p53 are significant predictors of poor responses to various immunotherapy-based treatment options (Table 3). Clinically, p53-mutant diffuse large B cell lymphoma (DLBCL) demonstrates significantly worse outcomes when treated with anti-CD19 CAR-T therapy, showing a complete response (CR) rate of 34%, markedly lower than the 65% CR rate observed in p53-WT patients [71]. Moreover, the 1-year overall survival (OS) rate for patients with *TP53* alterations is 44% compared to 76% for those without such alterations. Progression-free survival (PFS) is also shorter in the p53-altered group. Transcriptomic analysis showed alterations in interferon and apoptosis pathways, which are crucial for the cytotoxic effects of CAR-T cells [71].

p53 mutation is also a poor prognostic marker of allogeneic hematopoietic stem cell transplantation (allo-HSCT). HSCT remains the only curative treatment in many blood malignancies and the first therapeutic option for high-risk leukemia patients. HSCT is known to eliminate leukemia cells by the GvL effect by which reconstituted donor immune cells kill host leukemia cells. Relapse after HSCT is more common in p53-mutant patients, with a three-year OS rate as low as 10% in AML patients after HSCT [72,73]. *TP53* status is also an independent predictor of poor survival for myelodysplastic syndrome (MDS) patients treated with stem cell transplantation [74].

p53 mutations may significantly influence the response to immune checkpoint blockade (ICB) therapies. In gastric cancer, patients treated with nivolumab exhibit higher objective response rates in p53-WT patients compared to those with p53 mutations [75]. Similarly, worse survival outcomes are observed in p53-mutant patients treated with atezolizumab in non-small cell lung cancer (NSCLC) compared to patients without such mutations [76,77]. This impact of ICB is also evident in patients treated with pembrolizumab, where those with p53 truncating mutations have a shorter median survival compared to p53-WT patients, while those with p53 missense mutations show less clear but potentially negative impacts on survival [78]. Additionally, Kim et al. reported the poor immunotherapy response in p53-mutant metastatic solid tumors [79].

Contradictory reports, however, showed that mutant p53 can be more immunogenic compared to WT p53. For instance, the CD123 × CD3 bispecific antibody flotetuzumab demonstrated higher objective response rates in p53-mutant cases than in p53-WT cases, attributed to higher immune infiltration and IFN-gamma responses [80].

## 6. Strategies Enhancing the Efficacy of Immune Response by Targeting p53

**Figure 4:**
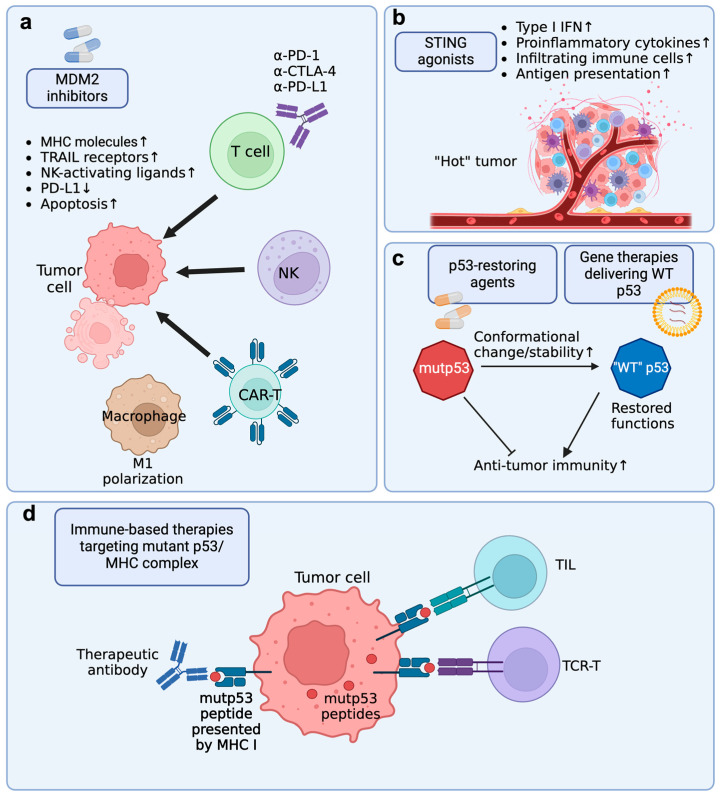
Strategies to enhance immune response against p53-WT and -mutant tumors. (**a**) MDM2 inhibitors can synergize with immunotherapies, such as ICB, immune cell adoptive transfer, and HSCT, by increasing the immunogenicity of tumor cells and polarizing macrophages in the TME into the proinflammatory M1 phenotype. (**b**) STING agonists have the potential to boost anti-tumor immunity against p53-mutant tumors and sensitize them to immunotherapies. (**c**) Novel p53-restoring small molecules and gene therapies delivering WT p53 can restore the function of mutant p53, making tumors susceptible to immune attack. (**d**) Immune-based therapies, such as TIL, TCR-T, and therapeutic antibodies, can kill p53-mutant tumor cells by recognizing mutant p53 peptides presented by MHC I on the tumor surface.

Given the role of p53 in regulating immune response, activating or restoring p53 functions is expected to enhance the anti-tumor immunity in cancers. In fact, p53-activating/restoring agents combined with various immunotherapies have been shown to achieve a synergy in treating multiple cancers in preclinical and clinical studies. One class of p53-activating agents is MDM2 inhibitors. Under normal conditions, p53 levels are kept low by MDM2, which binds to p53, promoting its ubiquitination and proteasomal degradation [81]. By inhibiting the interaction between MDM2 and p53, MDM2 inhibitors prevent the ubiquitination and subsequent degradation of p53, leading to the stabilization and accumulation of p53 protein in the cell. MDM2 inhibitors are being studied in combination with other therapies, such as chemotherapy and immune therapies, to enhance their efficacy and overcome resistance mechanisms. However, despite their promise in p53-WT cancers, most MDM2 inhibitors are not able to restore the functions of mutant p53. Therefore, some small molecules have been developed in recent years to restore the WT-like functions and structure in mutant p53, serving as promising candidates for treating p53-mutant cancers. It is noteworthy that, over the past decade, these small molecule p53-targeted therapies have experienced the most rapid growth among different p53-targeted therapies [82]. Despite this, other p53-targeted therapies, such as gene therapies and immune-based therapies, also gained huge interest in overcoming p53 mutation or deletion. In this section, we attempt to summarize the progress in targeting MDM2 to potentiate immunotherapies and discuss the potential of using STING agonists, p53-restoring agents, gene therapies, and immune-based therapies in targeting immune evasion in p53-mutant cancers (Figure 4).

### 6.1. MDM2 Inhibitor + Immune Checkpoint Blockade (ICB)

ICB has achieved success in treating multiple malignancies, such as lung cancer, triple-negative breast cancer, melanoma, and gastrointestinal cancers [83]. However, the response rate of ICB remains low. Multiple studies have shown that MDM2 inhibitors achieve a synergy with ICB in treating cancers (Figure 4a). In a pre-clinical study using syngeneic mouse lung cancer models, Zhu et al. found that tumor immunogenicity and response to ICB are dependent on p53 status, partially due to reduced antigen presentation [78]. Stabilizing p53 by MDM2 inhibitor Nutlin-3a increased T cell cytotoxicity and the efficacy of ICB. Another MDM2 inhibitor, APG-115, was also shown to enhance the anti-tumor activity of anti-PD-1 therapy in syngeneic mouse models [84]. Surprisingly, in the same study, APG-115 also enhanced anti-PD-1 therapy in treating p53-mutant and p53-null tumors. This is unexpected as MDM2 inhibitors are thought to induce apoptosis only in p53-WT tumors. This suggests that APG-115 might have tumor-independent effects, such as impacts on the tumor microenvironment. Mechanistic studies showed that the drug reprogramed immunosuppressive M2 macrophages into the proinflammatory M1 phenotype and increased cytotoxic CD8+ T cells in the tumors. Consistent with this finding, Guo et al. showed that Nutlin-3a reversed immunosuppression in the TME to induce systemic anti-tumor immunity and immune regression [58]. In another study, MDM2 inhibitor APR-246 was shown to counteract GOF immune evasive mechanisms by regulating IFN expression, promoting CD4+ T cell infiltration, and slowing T cell exhaustion in the TME [16]. These pre-clinical findings provide rationale for moving the combination therapy into clinical trials. A phase II clinical trial (NCT03611868) showed that APG-115 in combination with pembrolizumab was well-tolerated and had preliminary anti-tumor activity in multiple tumor types [85].

### 6.2. MDM2 Inhibitor + Immune Cell Adoptive Transfer Therapy

We have introduced that p53 can upregulate the expression of ligands for NK activating receptors, such as ULBP1/2, PVR, and Nectin-2 [42,56]. Therefore, MDM2 inhibitors might have a synergy with NK adoptive transfer therapy. Indeed, MDM2 inhibitors RITA and Nutlin-3a were shown to increase NK cytotoxicity in vitro and in vivo [42,56], which might be used for NK cell-based immunotherapy. Another study showed that Nutlin-3a effectively sensitized neuroblastoma cells to DNAM-1 CAR-NK cells by upregulating DNAM-1 ligands [86].

### 6.3. MDM2 Inhibitors + HSCT

Many patients suffer from relapse after HSCT [87]. Targeting p53 might be a potential way to prevent relapse after HSCT by activating anti-tumor immunity. In mouse models, MDM2 inhibitors successfully prolonged the survival of mice after HSCT by three converging mechanisms: MHC-II upregulation, TRAIL-R1/2, and direct activation of T cells [49]. This study suggested that giving MDM2 inhibitors to AML patients after HSCT might be a promising strategy to enhance anti-leukemia immunity and prevent relapse.

### 6.4. STING Agonists + Immunotherapies

Due to the importance of the STING pathway in anti-tumor immunity, various STING agonists have been developed to treat cancers [88]. Accumulating evidence has shown that p53-mutant tumors have reduced STING signaling and are immunologically “cold”, which underlies the immune evasion of the p53-mutant tumors [39,44]. Therefore, reactivating the STING pathway may be a potential way to restore the anti-tumor immunity against p53-mutant tumors. Putting STING back to p53-mutant blood cancers was shown to have a synergy with BH3 mimetics in p53-mutant cells and prolong the survival of tumor-bearing mice [89]. Therefore, it is also possible that STING agonists can enhance the apoptotic priming and immunogenicity of p53-mutant tumors, which make the malignant cells more responsive to immunotherapies such as ICB and CAR-T (Figure 4b).

### 6.5. Targeting Immune Evasion of p53-Mutant Cancers by p53-Restoring Agents

Mutant p53 had long been considered undruggable. Although MDM2 inhibitors can induce apoptosis in p53-WT cancers, their effect in p53-mutant cancers is limited. However, small molecules that restore the structure and functionality of mutant p53 give hope to enhance the immune response to p53-mutant cancers (Figure 4c). In a phase 1b clinical study, Eprenetapopt (APR-246) was combined with pembrolizumab in patients with advanced/metastatic solid tumors (NCT04383938) [90]. Eprenetapopt is a first-in-class p53-reactivating small molecule that covalently modifies cysteine residues in mutant p53, leading to restoration of WT p53 protein function. Overall, the combination of eprenetapopt and pembrolizumab was well-tolerated and showed clinical activity in heavily treated patients. Further clinical studies are needed to evaluate the anti-tumor activity of this therapy and determine responsive tumor subsets.

### 6.6. Restoring Function of Mutant p53 with Gene Therapies

The effectiveness of small molecules in reactivating mutant p53 is questionable [91]. An alternative approach to restoring p53 function involves delivering the WT *TP53* gene into tumor cells (Figure 4c). Various vectors have been explored for this purpose. One promising example is SGT-53, a cationic liposome complex encapsulating WT p53 cDNA. In a pre-clinical study, SGT-53 was shown to restore anti-tumor immunity and overcome tumor resistance to a checkpoint inhibitor [92]. In a phase II trial, SGT-53 combined with gemcitabine/nab-paclitaxel demonstrated encouraging results in treating metastatic pancreatic cancer [93]. mRNA nanoparticles have emerged as a promising platform for gene therapies, particularly following their success in COVID-19 vaccines. Synthetic mRNA nanoparticles could restore p53 expression and sensitize p53-deficient cancers to mTOR inhibition [94].

Although the concept of gene therapies seems promising, several challenges remain. (1) Can the gene delivery systems reach out to all tumor cells? If not, some p53-mutant or -null cells can remain resistant to anti-tumor immunity and therapies. (2) Mutant p53 can interfere with WT p53 in a dominant-negative manner [9]. Therefore, reintroduced WT p53 may not fully regain its function in cells harboring p53 mutations.

### 6.7. Targeting Mutant p53 by Immune-Based Therapies

Mutant p53 is an intracellular protein and is out of reach for immune cells and antibodies. However, it can be degraded into peptides and presented by MHC on the cell surface, making it targetable by T cells. Thus, missense mutant p53 may become a neoantigen and a potential target for immune-based therapies (Figure 4d). Tumor-infiltrating lymphocytes (TILs) isolated from p53-mutant tumors have been shown to recognize mutant p53 as a neoantigen, suggesting that mutant p53 is immunogenic [20,95]. It is, therefore, possible to use TCR-T or TIL therapies to treat p53-mutant cancers.

Kim et al. successfully generated a library of 39 TCRs that recognize tumor cells in a *TP53* mutation- and human leucocyte antigen (HLA)-specific manner [96]. They then treated 12 patients with TILs that were naturally reactive against p53 mutations. However, the efficacy was modest, with only two partial responses among twelve patients. They also treated one patient with TCR-T comprising autologous T cells transduced with an allogeneic HLA-A_02–restricted TCR specific for p53^R175H^, resulting in a tumor regression (−55%) over 6 months.

Bispecific T cell engagers have also become more and more popular in recent years. Hsiue et al. developed a bispecific antibody that targets p53^R175H^ presented by HLA-A*02:01 on one arm and CD3 on the other arm to engage T cells to kill tumor cells [97]. This strategy was effective even for killing tumor cells with low antigen density on their surface.

However, there are limitations to targeting p53 neoantigens. Although T cells can recognize tumor cells carrying p53 mutations, they may be unable to kill the tumor cells due to the immunosuppressive mechanisms of mutant p53, as discussed in previous sections. This was observed in p53-mutant large B cell lymphomas, which express CD19 antigen but remain insensitive to CD19-CAR-T therapy [71]. Combining TCR-T, TIL, or bispecific antibodies with ICB or other therapies could potentially improve outcomes by counteracting the immune-evasive mechanisms of p53-mutant tumors.

## 7. Discussion

Despite advancements in cancer treatment over the past decades, the effective treatment of p53-mutant tumors remains a formidable challenge. *TP53* mutation or deletion, which renders the tumor suppressor p53 inactive, was once considered completely undruggable due to the immense difficulty of targeting cancer cells carrying these p53 mutations or deletions. Historically, the primary focus of p53 has been centered on its role in cell cycle arrest, apoptosis, and senescence, all of which are critical for tumor suppression. However, the significance of p53 in regulating the immune response has been underappreciated. In this review, we have summarized up-to-date evidence from both basic research and clinical studies, which demonstrate the immune evasive properties of p53-mutant cancers. We have also reviewed the potential mechanisms by which *TP53* mutations facilitate immune evasion. These mechanisms include alterations in antigen presentation, modulation of immune checkpoints, alterations in the cancer cell secretome, modulation of NK cell activation, suppression of phagocytosis by macrophages, and changes in the tumor microenvironment that foster immunosuppression. The studies about the mechanisms are crucial for developing new therapeutic strategies.

Based on these findings, combinatorial therapies are currently under investigation to enhance current immunotherapies or to overcome the immune evasion mechanisms associated with *TP53* mutations. These therapies include the use of p53-reactivating agents in combination with ICB, HSCT, and cellular immunotherapies. Given the suppression of the cGAS-STING pathway by mutant p53, we believe that STING agonists should be further evaluated for the potential to restore the immune response in p53-mutant cancers. Future studies are also needed to rigorously assess the efficacy and safety of these combinatorial therapies.

Although this review mainly discusses the alterations of p53 in cancer cells, the p53 status of immune cells may also modulate their respective immune functions. Activating or reactivating p53 in non-tumor cells in the TME also represents a compelling strategy to counteract immunosuppression [98]. p53 mutations are identified in clonal hematopoiesis, which means these mutations can occur in hematopoietic stem cells and immune cells differentiated from these stem cells without overt malignancy [99]. However, whether p53 mutations in the immune compartment are associated with specific functional changes and disease outcome remains to be determined. Additionally, whether p53 mutations in pre-malignant clones promote malignant transformation via immune evasion remains to be studied.

## 8. Conclusions

Despite continuous advances in treating p53-mutant cancers, significant challenges remain, and further efforts are needed to develop novel compounds targeting these malignancies. p53-targeted drugs are unlikely to be effective as monotherapies, making it crucial to explore combination strategies that include p53-targeted therapies alongside immunotherapies, targeted therapies (e.g., BCL-2 inhibitors, SMAC mimetics, STING agonists), and others. In our view, genome-wide CRISPR screening offers a powerful approach for identifying mechanisms of immune evasion and potential therapeutic targets in p53-mutant tumors. Unlike transcriptomics and proteomics, CRISPR screens provide direct functional insights by loss-of-function or gain-of-function genetic manipulation in cells. These screens can be applied to both tumor and immune cell compartments, allowing for complete gene disruption, leading to clearer phenotypic outcomes. Additionally, drug screening is another strategy for discovering molecules that can be combined with immunotherapies to target p53-mutant cells.

In summary, while the role of p53 in cancer immunity is complex, the insights gained from recent studies provide a foundation for developing new therapeutic strategies. Translating these findings into effective treatments for patients with p53-mutant cancers remains a critical goal. Ongoing studies and clinical trials will be pivotal in bringing these innovative therapies from the laboratory to the clinic, ultimately improving outcomes and providing new hope for patients battling these challenging forms of cancer.

## Figures and Tables

**Table 1:** Common hotspot mutations in p53 and their respective effects.

Mutation Type	Location	Amino Acid Change	Phenotypic Effect	LOF/GOF *	Examples of Associated Cancers	Citations
Missense	Exon 5	R175H	Impaired DNA-binding ability, induced genetic instability	LOF	Breast, Lung, Ovarian	[21]
Missense	Exon 6	Y220C	Altered protein conformation and DNA-binding ability	LOF	Breast, Lung, Head and Neck	[20,22]
Missense	Exon 7	G245S	Altered DNA-binding domain	LOF	Ovarian, Breast, Lung	[23]
Missense	Exon 7	G245D	Disrupted DNA-binding domain	LOF	Colorectal, Breast, Ovarian	[24]
Missense	Exon 8	R248L	Reduced DNA-binding capacity	LOF	Various Cancers	[25]
Missense	Exon 8	R248Q	Reduced DNA-binding capacity	LOF	Ovarian, Esophageal, Colorectal	[9]
Missense	Exon 8	R248W	Reduced DNA-binding capacity	LOF and GOF	Breast, Colorectal, Pancreatic	[26]
Missense	Exon 8	R249S	Reduced DNA-binding capacity	LOF and GOF	Liver, Ovarian, Lung	[27]
Missense	Exon 8	R273C	Disrupted DNA-binding domain	LOF	Bladder, Lung, Colorectal	[28]
Missense	Exon 8	R273L	Alters DNA-binding domain	LOF and GOF	Various Cancers	[29]
Missense	Exon 10	R282W	Disruption of tetramerization and DNA-binding	LOF	Sarcoma, Colon, Brain	[30]

* LOF: loss of function; GOF: gain of function.

**Table 2:** *TP53* mutation types and their immunoregulatory effects.

Mutation Type	Immunoregulatory Effects	Affected Immune Cells	Tumor Type	Cell Line or Primary Tumor	References
Deletion	Reduced ERAP1 and MHC 1	T cells	Colon cancer	HCT116	[36]
	Upregulation of PD-L1	T cells	Colon cancer, lung cancer	HCT116, H1299	[37]
	Increased release of extracellular vesicles carrying PD-L1	Macrophages	B cell malignancies	Patient primary tumors, Eμ-TCL1	[38]
	Reduced cGAS/STING activation	Various immune cells	Lung cancer, colon cancer	A549, H1299, CT26	[39]
R172H (mouse)	Increased M2 polarization of macrophages caused by upregulation of CSF1 in mutant tumor cells	Macrophages	Esophageal squamous cell carcinoma	Chemically induced primary tumor in mice	[40]
	Increased release of CXCL2, which causes neutrophil infiltration	Neutrophils	Pancreatic ductal adenocarcinoma	Primary murine tumor	[41]
R175H	Reduced ERAP1 and MHC 1	T cells	Colon cancer	HCT116	[36]
	Reduced ligands for NKG2D	NK cells	Lung cancer	H1299	[42]
G245D	Reduced ERAP1 and MHC 1	T cells	Colon cancer	HCT116	[36]
R248W	Reprogramming of macrophages into M2 phenotype	TME	Colon cancer	HCT116	[43]
R249S (mouse)	Reduced cGAS/STING activation	Various immune cells	Lung cancer	4T1	[44]
R273C	Reduced ERAP1 and MHC 1	T cells	Colon cancer	HCT116	[36]
R280T	Reduced ERAP1 and MHC 1	T cells	Colon cancer	HCT116	[36]

**Table 3:** A summary of clinical studies that showed correlation between p53 mutations and response to immunotherapies.

Cancer Type	Immunotherapy	Findings	References
DLBCL	Anti-CD19 CAR-T	p53 alterations are associated with lower OR rate, shorter OS, and shorter PFS	[71]
AML, MDS	Hematopoietic stem cell transplantation (HSCT)	p53 mutations associated with lower 3-year RFS	[72,73,74]
Solid tumors (gastric cancer, colorectal cancer, breast cancer, NSCLC, etc.)	Immune checkpoint blockade (ICB)	p53 mutations correlate with poor efficacy of ICB	[75,76,77,78,79]
AML	Flotetuzumab (CD123 × CD3 bispecific)	Higher objective response rates in *TP53*-mutated cases	[80]
